# Current Treatment Landscape for Early Triple-Negative Breast Cancer (TNBC)

**DOI:** 10.3390/jcm12041524

**Published:** 2023-02-15

**Authors:** Jieun Lee

**Affiliations:** 1 Division of Medical Oncology, Department of Internal Medicine, Seoul St. Mary’s Hospital, College of Medicine, The Catholic University of Korea, Seoul 06591, Republic of Korea; befamiliar@catholic.ac.kr; Tel./Fax: +82-2-2258-6048; 2 Cancer Research Institute, The Catholic University of Korea, Seoul 06591, Republic of Korea

**Keywords:** triple-negative breast cancer, neoadjuvant chemotherapy, immune checkpoint inhibitor

## Abstract

Triple-negative breast cancer (TNBC) accounts for 15–20% of all breast cancers and is characterized by an aggressive nature and a high rate of recurrence despite neoadjuvant and adjuvant chemotherapy. Although novel agents are constantly being introduced for the treatment of breast cancer, conventional cytotoxic chemotherapy based on anthracyclines and taxanes is the mainstay treatment option for TNBC. Based on CTNeoBC pooled analysis data, the achievement of pathologic CR (pCR) in TNBC is directly linked to improved survival outcomes. Therefore, the treatment paradigm for early TNBC has shifted to neoadjuvant treatment, and the escalation of neoadjuvant chemotherapy to improve the pCR rate and the addition of post-neoadjuvant chemotherapy to control the residual disease have been investigated. In this article, we review the current treatment landscape for early TNBC, from standard cytotoxic chemotherapy to recent data on immune checkpoint inhibitors, capecitabine, and olaparib.

## 1. Introduction

Breast cancer is the most commonly diagnosed cancer worldwide [1] and the most common solid cancer in Korean female cancer patients [2]. Breast cancer is classified according to the expression of the estrogen receptor (ER), the progesterone receptor (PR), and human epidermal growth factor-2 (HER2) receptors based on immunohistochemical staining [3,4]. Breast cancers presenting with the absence of ER, PR, and HER2 are subtyped as triple-negative breast cancer (TNBC) and account for 15–20% of all breast cancers [5]. TNBC is more prevalent in younger patients and may harbor germline mutations in the pathogenic breast cancer gene 1 (BRCA1) or breast cancer gene 2 (BRCA2) [5]. 

The traditional treatment guidelines for early TNBC are based on surgery and postoperative adjuvant chemotherapy for the prevention of disease recurrence [6]. If a patient presents with inoperable, locally advanced breast cancer, neoadjuvant chemotherapy is considered to reduce tumor size and increase the possibility of breast-conserving surgery [7,8,9]. Compared to adjuvant treatment, administration of preoperative systemic treatment for breast cancer was not associated with improvement in disease-free survival (DFS) or overall survival (OS) [8,10]. However, there was a survival benefit in patients who achieved a pathologic complete response (pCR) after neoadjuvant treatment when compared to patients who showed residual disease [11]. The prognostic value of pCR was validated in CTNeoBC pooled analysis, demonstrating that pCR was associated with improved survival outcomes, especially in TNBC and HER2-positive breast cancer [12]. 

The development of novel agents such as HER2-targeting monoclonal antibodies, antibody–drug conjugates (ADC), and cyclin-dependent kinase 4/6 (CDK4/6) inhibitors has dramatically improved the survival outcome of hormone receptor (HR)-positive breast cancer and HER2-positive breast cancer [13,14]. However, TNBC only showed marginal improvements in survival outcomes due to its heterogeneous genomic landscape and lack of targetable molecular alterations [15,16]. Conventional cytotoxic chemotherapy is the mainstay of neoadjuvant and adjuvant treatment for early TNBC. Although the development and application of novel agents for TNBC is relatively slow, immune checkpoint inhibitors such as pembrolizumab and poly (ADP-ribose) polymerase (PARP) inhibitors such as olaparib have shown definite survival benefits in recent years. In this article, we review the current treatment guidelines and novel agents for early TNBC and outline future prospects of treatment patterns regarding escalation and de-escalation of systemic treatment for early TNBC.

### 1.1. Classical Adjuvant Treatment for TNBC

Adjuvant chemotherapy is recommended in TNBC patients who present with a tumor size of more than 1.0 cm and is considered in patients with a tumor size between 0.5 cm and 1.0 cm, irrespective of nodal involvement [17]. Patients with TNBC presenting with nodal involvement are definite candidates for adjuvant chemotherapy, irrespective of tumor size [17]. Adjuvant chemotherapy showed a consistent survival benefit with improved DFS in breast cancer [18] and has shown a greater survival benefit in TNBC than in hormone receptor (HR)-positive breast cancer [19]. Traditional adjuvant chemotherapy is based on anthracyclines, taxanes, and alkylating agents. Based on the EBCTCG 2012 meta-analysis, the addition of taxanes to anthracycline resulted in reduced cancer recurrence compared to that in the anthracycline-only group [18]. When anthracycline and taxane are both administered as adjuvant chemotherapy, dose-dense administration of anthracycline with G-CSF support has shown a greater survival benefit compared to the 3-weekly anthracycline group [20]. When taxane is administered, 3-weekly docetaxel and paclitaxel can be considered in the adjuvant setting [21]. In a subgroup analysis, weekly paclitaxel showed the most improved survival outcome and is a preferred option for TNBC [21]. In breast cancer patients with tumor sizes larger than 1.0 cm and smaller than 7.0 cm, docetaxel combined with cyclophosphamide (TC) showed favorable DFS compared to doxorubicin with cyclophosphamide (AC) in an adjuvant setting. TC showed favorable DFS compared to AC in all subgroups and can be considered as an option, as anthracycline can be spared and the risk of cardiotoxicity can be minimized [22]. Adjuvant docetaxel combined with anthracycline and cyclophosphamide (TAC) was compared with 5-FU + anthracycline + cyclophosphamide (FAC) in breast cancer. Irrespective of hormone receptor status or nodal involvement, TAC showed superior DFS compared to FAC [23], and an OS benefit was proven in the node-positive breast cancer subgroup [24]. TAC was compared to AC followed by taxane in the BCIRB005 trial, and there were no survival differences between the two regimens. The choice of the adjuvant regimen was carefully considered based on the toxicities of each regimen [25]. 

### 1.2. The Role of Platinum in Adjuvant Treatment for TNBC

Platinum agents inhibit DNA synthesis by forming cross-links with DNA, leading to cancer cell apoptosis in malignancies that harbor defective DNA repair mechanisms [26]. Patients harboring BRCA1/2 mutations show homologous recombination deficiency (HRD) and are susceptible to DNA repair damage when platinum agents are used [27]. Some patients with sporadic TNBC who do not harbor BRCA1/2 mutations show similar defects in the DNA repair mechanism, similar to BRCA1/2 mutant TNBC, called *BRCAness* [28]. Although not all patients with TNBC harbor HRD, there is a certain chance of BRCAness in sporadic TNBC. Based on the probability of BRCAness, platinum agents are preferred for the treatment of TNBC. 

Some single-center retrospective studies have analyzed the role of adjuvant platinum combined with standard anthracycline- and taxane-based regimens, with no confirmed clinical benefit [29,30]. However, recently conducted phase II and III trials have shown non-inferiority or superiority of platinum-containing adjuvant regimens over standard adjuvant regimens [31,32]. Owing to the small number of prospective trials, the benefit of adjuvant platinum-based regimens is unclear and needs to be validated by prospective ongoing adjuvant trials (Table 1).

### 1.3. Neoadjuvant Treatment for TNBC

In stage II or III TNBC, neoadjuvant chemotherapy is preferred based on various treatment guidelines [6,17]. The survival outcomes of patients receiving neoadjuvant or adjuvant chemotherapy after surgical resection are not statistically different, and neoadjuvant treatment is traditionally associated with an increased rate of local control, thereby guiding breast-conserving surgery with organ preservation [11,33]. As the development of neoadjuvant treatment is rapidly progressing, pathologic complete response (pCR), defined as the absence of malignant tumor cells in the breast and axillary lymph nodes, is frequently observed after surgical resection. Based on CTNeoBC pooled analysis, achievement of pCR is associated with improved survival outcomes, and neoadjuvant chemotherapy has evolved with the incorporation of novel regimens [12]. Similar to standard adjuvant treatment, traditional neoadjuvant chemotherapy is also based on anthracyclines and taxanes, and a dose-dense regimen is preferred in neoadjuvant settings based on improved DFS and OS proven in a meta-analysis [20]. Historically, anthracycline is usually administered prior to taxane administration because of the prior establishment before the introduction of taxane to standard treatment. Based on literature reviews, there were no differences in DFS, OS, and pCR rates according to the sequence of taxane administration before or after anthracycline treatment [34]. Considering that there was no significant difference in survival outcome according to the sequence of taxane administration, the current standard practice of delivering anthracycline first is maintained in most institutions. 

### 1.4. Addition of Platinum during Neoadjuvant Chemotherapy

To increase the pCR rate during neoadjuvant chemotherapy, the escalation of neoadjuvant treatment based on a combination regimen with platinum has been the focus in recent years. The combination of platinum and conventional taxane and anthracycline regimens has improved the pCR rate from approximately 35% to over 50% in TNBC [35,36,37]. The meta-analysis also revealed a similar survival benefit of combining platinum with taxane and anthracycline in patients with TNBC. The combination of platinum for neoadjuvant chemotherapy was based on the rationale that sporadic TNBC may show *BRCAness* and a good response to platinum [27,28]. Contrary to expectations, the combination of platinum showed the greatest benefit in sporadic TNBC patients who were gBRCA wild type, and the gBRCA mutant patient subgroup showed only a marginal benefit [37,38,39]. Previous clinical trials have focused on increasing the pCR rate, but the prolongation of survival is not fully validated, and a longer follow-up is warranted at present. Currently, a combination of platinum is recommended for selected patients, such as those who need adequate local control before surgical resection [17]. Currently, ongoing prospective neoadjuvant trials covering platinum combinations, such as the phase III PEARLY trial (NCT02441993), may suggest a more concrete role for platinum in neoadjuvant settings, and the long-term outcome of combining platinum with standard neoadjuvant treatment needs to be validated in the future. 

### 1.5. Role of an Immune Checkpoint Inhibitor in Neoadjuvant Setting

As pembrolizumab and atezolizumab have shown PFS benefits in phase III trials [40,41], the role of immune checkpoint inhibitors (ICI) has expanded into neoadjuvant settings. Although atezolizumab showed conflicting or disappointing results in advanced or early TNBC [42,43], pembrolizumab showed consistent PFS and OS benefits in advanced TNBC [40,44]. Furthermore, a combination of pembrolizumab with paclitaxel–carboplatin followed by anthracycline showed an increase in the pCR rate and also improved event-free survival rate [45,46,47]. The pivotal KEYNOTE-522 trial has changed the treatment paradigm for stage II and III TNBC by introducing pembrolizumab as a standard treatment during neoadjuvant treatment. The KEYNOTE-522 trial evaluated the role of pembrolizumab (18 cycles, 200 mg every 3 weeks) combined with four cycles of carboplatin (3 weekly) and paclitaxel (weekly or 3 weekly), followed by anthracycline plus cyclophosphamide (3 weekly), which powered the co-primary endpoint of increased pCR and EFS compared to placebo with chemotherapy. The addition of pembrolizumab showed a 13.6% improvement in pCR (64.8% (95% confidence interval; CI = 59.9–69.5%) vs. 51.2% (95% CI = 44.1–58.3%)) and a 7.7% improvement in the 36-month EFS rate (84.5% (95% CI = 81.7–86.9%) vs. 76.8% (95% CI = 72.2–80.7%)), meeting the primary endpoint of the study [46,47]. The combination of pembrolizumab showed a benefit irrespective of PD-L1 status evaluated by the 22C3 pharmDx assay or lymph node involvement status. Although the follow-up duration was immature, there were trends toward superior OS in the pembrolizumab-treated population, and further follow-up of the data is warranted [46]. Positive data from KEYNOTE-522 have changed the standard treatment guidelines for neoadjuvant treatment in stage II-III TNBC [17]. However, there are some open questions when applying pembrolizumab for neoadjuvant treatment in the clinic. First, further studies are warranted to select patients who may benefit the most from the addition of pembrolizumab. Fatal immune-related adverse events may occur during neoadjuvant pembrolizumab treatment plus chemotherapy. Therefore, it is necessary to choose patients who may benefit most from pembrolizumab, but there is no established biomarker for selecting appropriate patients. Unlike advanced or metastatic TNBC [40], PD-L1 expression was not associated with an improved pCR rate or EFS in the KEYNOTE-522 trial. Other immune-related markers, such as tumor-infiltrating lymphocytes (TIL), are currently being investigated [48,49], and follow-up of these results is needed. Second, the backbone chemotherapy regimen of the KEYNOTE-522 trial consisted of three weekly paclitaxel–carboplatin cycles followed by anthracycline and cyclophosphamide. Considering that dose-dense neoadjuvant regimens show superior OS benefits in TNBC [50], the incorporation of a dose-dense regimen with pembrolizumab needs to be evaluated based on a prospective randomized trial. 

### 1.6. Post-Neoadjuvant Treatment for TNBC

#### 1.6.1. Post-Neoadjuvant Treatment in Patients with Residual Disease

As previously mentioned, recent advances in neoadjuvant chemotherapy have greatly affected the treatment of TNBC by increasing the pCR rate. As neoadjuvant treatment has become a standard treatment, clinicians have focused on patients who do not achieve pCR. Non-pCR patients show poor survival outcomes compared to pCR patients [12], and post-neoadjuvant treatment has been applied to non-pCR patients to achieve prolonged survival outcomes. The escalation of treatment in non-pCR patients has effectively prolonged survival outcomes, and there has been great success in this patient subgroup. 

The phase III CREATE-X trial enrolled patients who showed residual disease after neoadjuvant anthracycline and taxane treatments [51]. Among the total patient population, 32.2% of patients were classified as TNBC, and approximately 40% of patients were clinically diagnosed with stage IIIA or IIIB TNBC. Patients who received 6–8 cycles of adjuvant capecitabine showed superior DFS, fulfilling the primary endpoint (HR = 0.70, 95% CI = 0.53–0.92, *p* = 0.01). At the time of data analysis, the median OS was not reached, and capecitabine-treated patients showed better OS compared to the control arm (HR = 0.59, 95% CI = 0.39–0.90, *p* = 0.01). In the prespecified subgroup analysis, TNBC patients still showed superior DFS and OS (HR for recurrence = 0.58, 95% CI = 0.39–0.87; HR for death = 0.52, 95% CI = 0.30–0.90). Although capecitabine-treated patients showed a higher rate of adverse events (AEs), such as hand-foot syndrome, AEs were generally well manageable with the maintenance of relative dose intensity in more than 80% of enrolled patients. Trials conducted prior to CREATE-X failed to prove the positive survival benefit of capecitabine [52,53], but these conflicting data may result from the small number of TNBC patients enrolled in the study [51]. In the Finland Capecitabine Trial (FinXX), although the prolongation of DFS was not validated, there were significant improvements in OS in the capecitabine and docetaxel combination group [53]. Although the administration schedule of capecitabine was different, the SYSUCC-001 trial showed prolongation of DFS in patients who received 1 year of metronomic capecitabine after completion of adjuvant treatment [54], and the CBCSG010 trial showed that the combination of capecitabine with standard adjuvant treatment improved DFS and OS, meeting the primary endpoint [55]. 

At present, the CREATE-X trial is the first phase III trial validating the positive role of capecitabine in post-neoadjuvant treatment and is accepted as the standard treatment for TNBC patients who show residual disease after neoadjuvant chemotherapy [6,17]. Recently, a phase III ECOG-ACRIN EA1131 trial was conducted comparing four cycles of post-neoadjuvant platinum to the standard six cycles of post-neoadjuvant capecitabine in basal subtype TNBC patients presenting with residual disease after neoadjuvant chemotherapy [56]. After a median follow-up of 20 months, platinum showed inferior 3-year invasive DFS (iDFS) compared to capecitabine-treated patients (3-year iDFS, 42% vs. 49%, HR = 1.06, 95% CI = 0.62–1.81), and the trial was terminated early. This trial further strengthens the role of post-neoadjuvant capecitabine therapy in patients with non-pCR TNBC. 

The KEYNOTE-522 trial was started before adjuvant capecitabine was considered the standard adjuvant regimen in non-pCR TNBC patients. Therefore, adjuvant capecitabine was not allowed during the KEYNOTE-522 trial, and pembrolizumab was administered after the completion of neoadjuvant chemotherapy. Regardless of the pCR status, the pembrolizumab arm showed superior outcomes compared to the control arm. In the prespecified subgroup analysis, patients with residual disease showed improved EFS when treated with pembrolizumab compared to the control arm (3-year EFS, 67.4% vs. 56.8%). TNBC patients receiving capecitabine in the CREATE-X trial showed a 3-year DFS of 69.8% compared with 56.1% in the control group [51], which is similar to the 3-year EFS rate of KEYNOTE-522. However, this head-to-head comparison should be conducted with caution because platinum was not administered to patients who were enrolled in the CREATE-X trial. 

Among non-pCR patients enrolled in the KEYNOTE-522 trial, the residual cancer burden (RCB) score was analyzed, and administration of pembrolizumab showed the greatest benefit in the RCB score two patients, with a 3-year DFS of 75.7% compared to 55.9% in the control group (HR = 0.52, 95% CI = 0.32–0.82) [57]. However, although there were only a small number of patients to be analyzed, RCB-3 patients showed a poor 3-year EFS of approximately 30%, irrespective of pembrolizumab administration. These non-pCR patient populations require more intensive studies to improve their survival outcomes, and ongoing studies might provide an answer to this unmet need. The currently ongoing phase II MIRINAE study (NCT03756298) may provide some insight into the role of escalation of post-neoadjuvant treatment by combining ICI with capecitabine. In addition to the combination of capecitabine and ICI, the role of pembrolizumab monotherapy in patients presenting with residual disease is currently under investigation. The SWOG S1418/NRG BR006 trial, which enrolled patients who had residual disease after neoadjuvant chemotherapy and received pembrolizumab for 1 year, has completed patient accrual and will provide further insight into the role of adjuvant ICI in patients who present with residual disease after neoadjuvant chemotherapy [58].

There are also other clinical trials involving ICI, capecitabine, and new agents such as ADC for patients with residual disease after neoadjuvant treatment (Table 2).

#### 1.6.2. Post-Neoadjuvant Treatment in Pembrolizumab-Treated Patients with pCR

Patients who achieved pCR after neoadjuvant chemotherapy showed a good prognosis in a meta-analysis [12], and this was also verified in the KEYNOTE-522 trial. Patients who achieved pCR showed favorable outcomes, irrespective of pembrolizumab administration. The 3-year EFS rate was 94.4% in the pembrolizumab arm and 92.5% in the control arm (HR = 0.73, 95% CI = 0.39–1.36), with no statistical difference [47]. Considering that ICI administration infrequently results in the development of autoimmune-related AEs and also causes financial toxicities in certain circumstances, de-escalation of ICI can be considered in this subgroup of patients. In the phase II GeparNuevo trial, durvalumab was administered as part of neoadjuvant chemotherapy with nab-paclitaxel and dose-dense epirubicin or cyclophosphamide. Although durvalumab was not administered after surgery, patients who achieved pCR showed excellent survival outcomes (3-year iDFS rate, 95.5%) [59]. The phase II NeoPACT trial administered pembrolizumab with docetaxel and carboplatin for six cycles, and patients who achieved pCR showed good 2-year EFS (98% vs. 78%, *p* = 0.001), although pembrolizumab was not used after surgery [60]. The NeoTRIP trial evaluated the role of atezolizumab combined with neoadjuvant nab-paclitaxel plus carboplatin, with the primary endpoint of increased pCR in the atezolizumab arm [61]. Although the primary endpoint was not met, the NeoTRIP trial only administered atezolizumab in the neoadjuvant setting, and further EFS data are awaited, which might explain the role of de-escalating ICI in the adjuvant setting in patients who achieved pCR after neoadjuvant treatment. 

#### 1.6.3. Adjuvant Treatment in a Special Population: The gBRCA Mutant Patient Subgroup

gBRCA mutations have been detected in 10–15% of unselected TNBC patients [62]. Germline BRCA1 and BRCA2 mutant carriers have a 65% and 45% risk of developing breast cancer at the age of 70, respectively [63]. In metastatic gBRCA1/2 mutant breast cancer, olaparib and talazoparib have shown benefits for prolonging PFS [64,65] and are currently approved by the FDA.

The phase III OLYMPIA trial verified the role of adjuvant olaparib in HER2-negative gBRCA1/2 mutant high-risk breast cancer [66]. Enrolled TNBC patients who underwent upfront surgery were eligible if they were diagnosed with stage II disease or above. If a patient receives neoadjuvant chemotherapy, the residual disease should be confirmed in the surgical pathology specimens. ER-positive gBRCA1/2 mutations who underwent upfront surgery should present with at least four positive lymph node involvements. If neoadjuvant chemotherapy is administered, patients should have a residual disease with clinical and pathological staging (CPS) ER status and a histologic grade (EG) score of 3 or higher [67]. Patients were randomly assigned in a 1:1 ratio to receive adjuvant olaparib or placebo for 1 year. The olaparib arm showed excellent improvement in 3-year iDFS compared to the placebo arm (85.9% vs. 77.1%, HR = 0.58, 99.5% CI = 0.41–0.82, *p* < 0.001). Subsequent follow-up data demonstrated that 1 year of adjuvant olaparib also prolonged the 4-year OS rate to 89.8% compared to 86.4% in the control arm, with an absolute difference of 3.4% (HR = 0.68, 98.5% CI = 0.47–0.97, *p* = 0.009) [68]. During 3.5 years of median follow-up, two acute myeloid leukemia (AML) cases were diagnosed in the olaparib arm and three patients in the control arm, demonstrating no new safety signal of hematologic disorders. This absolute benefit of adjuvant olaparib in gBRCA1/2 mutant early breast cancer (EBC) has been reflected in treatment guidelines, and adjuvant olaparib is currently the standard treatment in gBRCA1/2 mutant EBC patients [17]. Considering this definite survival benefit of olaparib, gBRCA1/2 testing should be actively considered in TNBC patients regardless of a patient’s age. 

The CREATE-X, KEYNOTE-522, and OLYMPIA trials have all changed the treatment paradigm in early TNBC, and prior trials should be fully considered when choosing adjuvant treatment in gBRCA1/2 mutant patients. Careful decision-making is warranted, considering that the CREATE-X and KEYNOTE-522 trials did not consider gBRCA1/2 mutation status during the study. In gBRCA1/2 mutant patients who were pretreated with neoadjuvant chemotherapy without pembrolizumab, olaparib and capecitabine can both be options for adjuvant treatment for patients presenting with residual disease, considering that the survival benefit was proven in previous pivotal trials. Among these two agents, olaparib can be carefully considered over capecitabine for several reasons. First, an adjuvant PARP inhibitor for gBRCA1/2 mutant TNBC directly targets the DNA damage repair pathway and may show high sensitivity and act as a targeting agent in this population of patients [69]. Second, the adjuvant capecitabine trials did not consider BRCA mutations during the analysis. In addition to CREATE-X, the SYSUCC-001 and CBCSG010 trials did not consider gBRCA1/2 status during analysis, and the lack of sufficient data to consider capecitabine can have a role in gBRCA1/2 mutant patients [51,54,55]. Post hoc analysis of the FinXX trial demonstrated that combining capecitabine may be more beneficial in non-BRCA-like tumors than in BRCA-like tumors [70], and these data may also support the use of olaparib in gBRCA1/2 mutant TNBC patients showing residual disease after neoadjuvant chemotherapy. 

In gBRCA1/2 mutant patients who received neoadjuvant pembrolizumab, as in the KEYNOTE-522 trial, and who presented with residual disease, adjuvant pembrolizumab or olaparib can be considered as an option. Each agent can be used as a single agent, or a combination can be carefully considered as a novel option. Preclinical data suggest that PARP inhibitors can induce T-cell recruitment in the tumor microenvironment, thereby enhancing the efficacy of ICI [71]. A few clinical trials have evaluated the effect of PARP inhibitors combined with ICI in metastatic or neoadjuvant settings [72,73,74]. In prior trials, there were no safety issues related to combination treatments. However, the efficacy of PARP inhibitors plus ICI in the metastatic setting [72,73] was not superior to historical data from the EMBRACA and OlympiAD trials [64,65]. Currently, there are clinical trials ongoing to evaluate the role of a combination of ICI and PARP inhibitors. The phase II WJOG14020B (NCT05485766) trial is trying to verify the role of concomitant pembrolizumab and olaparib as neoadjuvant and adjuvant treatments in gBRCA mutant EBC patients. Analyzing the results of currently ongoing trials and further long-term follow-up is warranted to decide the future treatment. 

### 1.7. Future Directions and Biomarkers

Recent advances in incorporating ICI and novel agents have changed the treatment paradigm for early TNBC, and the treatment pattern has shifted to the escalation of chemotherapeutic agents based on standard neoadjuvant regimens. However, we also need to focus on the subpopulation that may have a good prognosis, and neoadjuvant chemotherapy can be considered to be de-escalated. In the KEYNOTE-522 trial, the placebo arm showed a 51.2% pCR rate, and patients presenting with pCR showed excellent survival outcomes, with a 3-year DFS rate of >90% [47]. Therefore, the investigation of novel biomarkers is important to classify patients who may have a good prognosis and de-escalate neoadjuvant chemotherapy with minimal toxicities. TIL is a promising biomarker to select patients who may show a good prognosis with de-escalation of treatment. In a registry-based retrospective analysis, young TNBC patients who were node-negative and presented with high TILs of 75% or more showed excellent long-term prognosis with a 15-year cumulative incidence of distant metastasis or death of 2.1% even if they did not receive chemotherapy [75]. A recently performed phase II NeoPACT trial also suggested that a higher stromal TIL level (≥30%) is associated with an increased pCR rate [60]. Utilizing liquid biopsies, such as circulating tumor DNA (ctDNA), can be a promising marker to select patients who may benefit from de-escalating neoadjuvant chemotherapy or escalating neoadjuvant or adjuvant treatment, and vice versa. Rapid clearance of ctDNA during neoadjuvant chemotherapy in early TNBC is associated with a high probability of achieving pCR [76]. In contrast, the detection of ctDNA after completion of neoadjuvant chemotherapy and surgery is associated with a higher rate of recurrence and a poorer prognosis [77]. The detection of ctDNA after definite surgery is related to the detection of minimal residual disease in TNBC, and the c-TRAK trial has been performed to investigate the role of upfront pembrolizumab [78]. Although the c-TRAK trial failed to prove the benefit of upfront ICI, biomarker-driven escalation treatment in this high-risk patient population is an important issue for further study. Clinical trials are ongoing to evaluate the role of escalation of post-neoadjuvant treatment in patients with detectable ctDNA (Table 3). 

## 2. Conclusions

**Figure 1:**
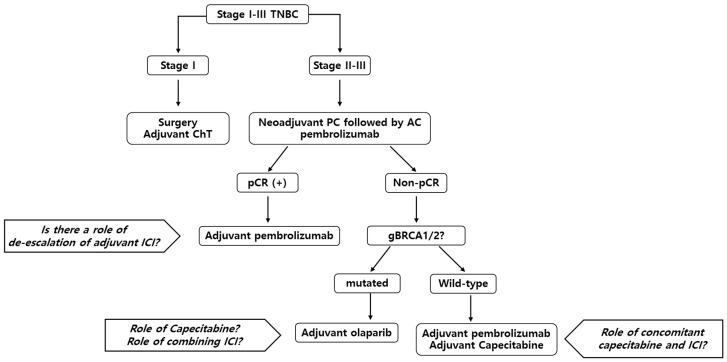
Suggested summarized systemic treatment flow for early TNBC patients. Abbreviations: TNBC, triple-negative breast cancer; ChT, chemotherapy; PC, paclitaxel + carboplatin; AC, anthracycline + cyclophosphamide; pCR, pathologic complete response; gBRCA, germline breast cancer gene; ICI, immune checkpoint inhibitor.

The standard treatment for early TNBC is neoadjuvant chemotherapy, followed by surgery. Based on the survival benefit of the KEYNOTE-522 trial, a combination of pembrolizumab with anthracycline, taxane, and carboplatin is currently the treatment of choice. After completion of neoadjuvant treatment, adjuvant treatment options can vary based on the presence of residual disease and gBRCA1/2 mutation status. In patients who present with pCR, completion of pembrolizumab treatment for a total of 1 year can be considered. However, several options can be considered for patients with residual disease. Other than the completion of pembrolizumab, capecitabine and olaparib should be considered according to the patient’s status. Currently, there is no evidence of a combination of ICI with capecitabine or olaparib, but there are ongoing clinical trials to determine the best treatment strategy for this patient population. The suggested treatment flow is described in Figure 1. Finally, biomarker-based studies are available to select patients with good prognoses and to de-escalate systemic treatment to minimize chemotherapy- or ICI-induced AEs and financial toxicities. 

## Figures and Tables

**Table 1:** Summary of ongoing trials investigating platinum in adjuvant setting.

Trial	Phase	No	Inclusion Criteria	Study Arm	Primary Endpoint
NRG-BR003 (NCT02488967)	III	782	node-positive or high-risk node-negative patients	AC followed by PC vs. AC followed by P	invasive DFS
PEARLY (NCT02441933)	III	840	stage II-III TNBC	Adjuvant AC followed by PC or TC vs. adjuvant AC followed by P or T	5-year DFS
TCTN (NCT02455141)	III	970	stage II-III TNBC	EC followed by PC or TC vs. EC followed by P or T	3-year DFS
NCT03876886	III	200	node-positive or high-risk node-negative TNBC with HRD	ddEC followed by PC ddEC followed by P	3-year DFS

AC, anthracycline + cyclophosphamide; PC, paclitaxel + carboplatin; P, paclitaxel; DFS, disease-free survival; TNBC, triple-negative breast cancer; TC, docetaxel + carboplatin; T, docetaxel; EC, epirubicin + cyclophosphamide; HRD, homologous recombination deficiency; ddEC, dose-dense epirubicin + cyclophosphamide.

**Table 2:** Summary of ongoing trials in patients with residual disease after neoadjuvant treatment.

Trial	Phase	No	Inclusion Criteria	Study Arm	Primary End Point
Chemotherapy-based					
TCTN (NCT02455141)	III	970	stage II–III TNBC	EC followed by PC or TC vs. EC followed by P or T	3-year DFS
NCT03876886	III	200	node positive or high risk node-negative TNBC with HRD	ddEC followed by PC vs. ddEC followed by P	3-year DFS
NCT04437160	II	286	TNBC with residual disease after platinum/taxane based NACT	EC vs. observation	Recurrence-free survival
NCT04297267	II	100	TNBC with residual disease anthracycline and paclitaxel allowed, platinum not permitted	gemcitabine + cisplatin for 4 cycles, single arm	3-year DFS
Immune-checkpoint-inhibitor-based treatment					
SWOG S1418/NRG BR-006 (NCT02954874)	III	1155	TNBC with residual disease after NACT *residual disease: ≥1 cm residual invasive carcinoma in the breast or positive micro- or macroscopic lymph nodes (ypN1mi-3)	adjuvant pembrolizumab for 1 year vs. observation	Invasive DFS
A-Brave (NCT02926196)	III	474	TNBC after neoadjuvant chemotherapy/adjuvant chemotherapy regardless of residual disease	adjuvant avelumab for 1 year vs. observation	5-year DFS
BreastImmune03 (NCT03818685)	II	95	TNBC with residual disease after NACT	adjuvant nivolumab + ipilimumab vs. adjuvant capecitabine	2-year DFS
MIRINAE (NCT03756298)	II	284	TNBC with residual disease after NACT *residual disease: ≥1 cm residual invasive carcinoma in the breast or macroscopically positive lymph nodes	adjuvant atezolizumab + capecitabine vs. adjuvant capecitabine	5-year DFS
OXEL (NCT03487666)	II	45	TNBC with residual disease after NACT *residual disease: ≥1 cm residual invasive carcinoma in the breast or macroscopically positive lymph nodes	nivolumab for 6 cycles capecitabine for 6 cycles nivolumab + capecitabine for 6 cycles	Changes in a peripheral immunoscore at week 6
Antibody-Drug conjugate					
SASCIA (NCT04595565)	III	1200	TNBC/HER2-negative breast cancer with residual disease after neoadjuvant chemotherapy -taxane; anthracyclines allowed; ICI allowed	sacituzumab govitecan for 8 cycles vs. capecitabine/carboplatin/cisplatin for 8 cycles or observation	Invasive DFS
ASCENT-05 (NCT05633654)	III	1514	TNBC with residual disease after NACT	sacituzumab govitecan + pembrolizumab for 8 cycles vs. pembrolizumab monotherapy for 8 cycles or pembrolizumab + capecitabine for 8 cycles	Invasive DFS
TROPION-breast 03 (NCT05629585)	III	1075	TNBC with residual disease after NACT	datopotamab deruxtecan for 8 cycles + durvalumab 9 cycles or datopotamab deruxtecan for 8 cycles vs. capecitabine for 8 cycles or pembrolizumab for 9 cycles (prior neoadjuvant pembrolizumab) capecitabine for 8 cycles + pembrolizumab for 9 cycles (prior neoadjuvant pembrolizumab)	Invasive DFS

TNBC, triple-negative breast cancer; EC, epirubicin + cyclophosphamide; TC, docetaxel + carboplatin; P, paclitaxel; T, docetaxel; DFS, disease-free survival; HRD, homologous repair deficiency; ddEC, dose-dense epirubicin + cyclophosphamide; NACT, neoadjuvant chemotherapy; HER2, human epidermal growth factor receptor 2; ICI, immune checkpoint inhibitor.

**Table 3:** Summary of ongoing trials according to biomarker.

Trial	Phase	No	Inclusion Criteria	Study Arm	Primary Endpoint
PERSEVERE (NCT04849364)	II	197	TNBC with residual disease after NACT *residual disease: ≥1 cm residual invasive carcinoma in the breast or macroscopically positive lymph nodes or RCB score II or III *allocating factor: positivity of ctDNA/presence of a genomic target	arm 1a (DNA repair target): talazoparib + capecitabine (closed) arm 1b (ICI target): atezolizumab + capecitabine arm 1c: (PI3K target): inavolisib + capecitabine, followed by atezolizumab arm 1d (DNA repair target + ICI): talazoparib + atezolizumab + capecitabine arm 2 (ctDNA+, no target): capecitabine or TPCarm 3 (ctDNA−): observation or capecitabine or TPC	DFS
ARTEMIS (NCT04803539)	II	260	stage II-III TNBC with positive ctDNA after curative surgery and/or adjuvant chemotherapy	capecitabine 650mg/m^2^ bid for 1 year capecitabine 650mg/m^2^ bid + apatinib + camrelizumab for 1 year	iDFS
Apollo (NCT04501523)	II	460	TNBC with or without residual disease, with ctDNA positive at baseline *arm A/B: TNBC with residual disease, ctDNA positive *arm C: TNBC with pCR, ctDNA positive	arm A: tislelizumab + capecitabine 600–750mg/m^2^ bid for 1 year arm B: capecitabine 600–750mg/m^2^ bid for 1 year arm C: capecitabine 600–750mg/m^2^ bid for 1 year arm D: observation	5-year DFS
ASPRIA (NCT04434040)	II	40	TNBC with residual disease after NACT positive ctDNA	single arm atezolizumab + sacituzumab govitecan for 6 cycles	rate of undetectable ctDNA after 6 cycles
ZEST (NCT04915755)	III	800	cohort 1: HER2-negative BC with somatic BRCA mutation cohort 2: TNBC with positive ctDNA	niraparib vs. observation * adjuvant capecitabine allowed, niraparib after completion of adjuvant capecitabine	DFS

TNBC, triple-negative breast cancer; NACT, neoadjuvant chemotherapy; DNA, deoxyribonucleic acid; ICI, immune checkpoint inhibitor; PI3K, phosphoinositide 3-kinases; DFS, disease-free survival; ctDNA, circulating tumor DNA; TPC, treatment of physician’s choice; iDFS, invasive disease-free survival; pCR, pathologic complete response.

## Data Availability

Not applicable.
